# Notes and News

**Published:** 1888-02-11

**Authors:** 


### Notes and News.

Collected and Communicated.

Mr. T. Dyer Edwardes has given a donation of £1,000 towards the reduction of the heavy mortgage debt on the Chelsea Hospital for Women.

At the annual meeting of the Blackheath and Charlton College Hospital, held in the lecture-hall of the Congregational Church, Blackheath, on the 28th ult., it was announced that the Earl of St. Germans had presented the site for the erection of a new hospital on his Kidbrooke estate.

A most interesting exhibition of Ducker's portable hospitals and houses is to be seen in Charles Street, Whitehall (opposite the India Office). A building capable of containing twelve beds can be carried on a single van, and can be erected in about an hour without the use of a single tool.

Mademoiselle Huchard has given £100 to provide spring beds for the patients in the Isle of Wight Infirmary. Above £232 has been received for the building fund, for the erection of nurses' bed-rooms and a new operating theatre; but it is estimated that these improvements will cost at least £600.

Mr. William Beer has been obliged, on account of impaired health, to resign the secretaryship of the Royal South Hants Infirmary, but will retain his office till a successor has been appointed to him. Mr. Beer is the senior official of the infirmary, having been connected with it for twenty-six years, during which time he has fulfilled his continually-augmenting duties with faithfulness and zeal.

The Chairman, at the quarterly meeting of the Governors of Grimsby Hospital, suggested that to reduce expenditure they should use the hospital only for accident cases. The present expenditure is now £1,600 a-year, and the actual subscriptions do not reach a third of that amount. We hope the good people of Grimsby will wake up to a sense of the usefulness of their hospital, and not permit it to be thus impaired through poverty.

It has been the custom of the Bath Board of Guardians to give an annual donation of ten guineas to the Bath Eye Infirmary, but recently they have talked of discontinuing it. Mr. Mason, the medical officer, therefore wrote to the board, telling of the work done at the infirmary, and pointing out to them that it did much to save the rates by preventing many who suffered from diseases of the eye from lapsing into hopeless blindness. The Guardians, on receipt of this letter, reconsidered the matter at their next meeting, and gave the donation as usual. An interesting incident arose out of this meeting. A former patient, who came a few years ago from the Bath Union to the Infirmary suffering from cataract and totally blind, had an operation performed which restored his sight, and is now earning his living. He wrote to Mr. Mason expressing his surprise that the Guardians should think of withholding their subscription, as in his case alone they had saved more than the amount of it every year, thanks to the work of the Infirmary. Another former patient shows her gratitude for the benefits she has received from the institution by sending every year a present of garments of her own making for the use of the patients.

A new ward has been added to the Liverpool Hospital for Women, for which Mrs. Lister has given the beds, but the committee cannot utilise it till the income of the hospital is increased.

Dr. Mapother, the new-elected president of the Dublin branch of the British Medical Association, delivered an address on the 25th ult. on "Our Hospitals," in which he made useful suggestions on various matters, in particular on the necessity of having hospitals for infectious diseases totally removed from general hospitals.

When the Chelmsford Infirmary was first established it was customary to charge patients 5*s.* a-week. This fee has now been reduced to 3*s.*; and the committee are anxious to lower it to 2*s.*, as a preliminary step to abolishing payment altogether. Even at present the committee often remit the fees when it seems necessary, but they would like to make free treatment the rule and not the exception.

The Nottingham Hospital for Women is in a far from prosperous condition. The subscriptions last year amounted to less than £200, and though the fees of paying patients enable the institution to keep going—though with a usefulness slightly diminished where it might well be enlarged—the banking account of the hospital shows a deficit of £454 6*s.* 5*d.* We should have expected better things from a town so important and energetic as Nottingham.

At the last meeting of the Norfolk and Norwich Hospital, Mr. G. H. Tuck spoke of the contemplated resignation by Sir Peter Eade, Mr. Crosse, and Mr. Cadge of their connexion with the Hospital, and expressed the desire the governors had to retain them as consulting physicians in reality—not merely in name. As a mark of the high appreciation in which the services of these three gentlemen are held, the governors propose allotting three beds in the hospital to each, to be filled with patients whom they shall recommend.

There are some bold—we will not say brave—people in the world. The following letter was read at the last meeting of the Westminster Memorial Cottage Hospital at Shaftesbury: "My dear Sir,—I shall feel obliged if you will be so good as to inform me why the Shaftesbury Cottage Hospital is not provisioned by tender, as is usual with such institutions. Since the Cottage Hospital is supported by all religious denominations, dissenters feel that it is only right that they should have an opportunity of supplying some of the provisions. I call attention to this anomalous state of things in regard to the provisioning of the institution, that it may be remedied. I fear that if the hospital continues to be provisioned as at present, the funds will suffer.—Yours faithfully, A. S. White, Wesleyan Minister." The chairman protested, on behalf of the Hospital Committee, that they had no thought of favouring one denomination above another in the matter of its custom; they had merely gone where they were best served. It came out in the course of the subsequent discussion of the matter, that Mr. White, who thus volunteered his advice about catering for the hospital, was not a subscriber to its funds; and Mr. H. C. Norton recorded, as a Wesleyan, his protest against the letter. Presumably Mr. White himself has no pecuniary interest in the advice he tendered, but is only looking after that of some member of his church. But it is not usual for him who ministers to any flock in spiritual things, to tout for temporal benefits for any individual of it. No doubt Mr. White's threat that "the funds would suffer," has an infinitesimal amount of truth in it. There are some people who will not lend to the Lord at a lower rate than a hundred per cent.; but, as a rule, their offerings are not so large that they would be missed. In any case, the half-crown of the grocer or butcher, who gives it only that he may get it back with interest, is not worth the bribe he demands.

THE following vacancies are announced this week, the amount of salary in each case being given after the name of the institution :—

**Resident Medical Officers.**

Bournemouth Friendly Societies Medical Association. Resident Medical Officer.—Salary £200 per annum, with fees.
Menston Asylum, near Leeds. Medical Superintendent.—£400 per annum, and residence.
Royal National Hospital for Consumption, Ventnor. Assistant Resident Medical Officer.
Royal Surrey County Hospital, Guildford. House Surgeon.—£80.
York Dispensary. Three Resident Medical Officers.—£130, with furnished apartments.

**Non-resident Medical Officers.**

Birmingham Borough Asylum. Clinical Assistant.
Bristol Royal Infirmary. Dental Surgeon.
Cancer Hospital, Brompton. Pathologist.—£80 for twelve months.
Forest Hill Provident Dispensary. Medical Officer.
National Orthopædic Hospital. Surgical Registrar and Anæsthetist.—Honorarium £20.
North-West London Hospital, Kentish Town Road. Assistant Physician.
Oldcastle Union. Medical Officer.—£135 per annum, and usual fees.
Royal Hospital for Diseases of the Chest, City Road. Assistant Physician.
Rutly Hill Asylum, Bromsgrove, Worcester. Clinical Assistant.
St. John's Hospital for Diseases of the Skin, Leicester Square. Two Assistant Medical Officers.
St. Mary's Hospital for Women and Children, Quay Street, Manchester. Honorary Surgeon.

**Nurses.**

Oswestry Cottage Hospital. Surgical Nurse.—£24.
R[illegible]herham Hospital. Assistant Nurse.
Huddersfield Union. Nurse and Assistant Nurse.—£30 and £20.
Cottage Hospital, Eltham, Kent. One.
Berkdale Nurses' Home, Crosby Road, East Southport. Trained Medical and Surgical. Several.

**Probationers.**

Royal National Hospital for Consumption, Ventnor. Several.—£10 first year.

---

DR. ARTHUR J. DAVIES has been appointed physician to the Royal Hospital for Diseases of the Chest, City Road, in the place of Dr. Pringle, who has resigned, and Mr. Walter T. Strugnell has been appointed senior house physician.

---

"LET Glasgow flourish !" Its inhabitants are not only rich but generous. The treasurer of the Royal Infirmary there had the satisfaction of announcing at the annual meeting that the receipts exceeded the expenditure by £14,884 12*s.* 1*d.* And yet Scottish parsimony is said to be proverbial !

---

THE *Bolton Chronicle* says: "An exceedingly handsome bequest was on Tuesday afternoon handed over to the hon. treasurer of the Bolton Infirmary (Mr. Alderman Musgrave, J.P.). A little over a year ago Miss Alice Lowe, of South Shore, Blackpool, and formerly of Rivington, died, leaving several handsome sums in aid of the working of useful associations with which she was well acquainted. The executors of Miss Lowe (Dr. Sharpe, South Shore, and R. Handley, Esq., Blackpool), accompanied by Mr. Joseph Harrison, South Shore, solicitor under the will, attended by appointment at the office of Mr. J. H. Bradbury, solicitor, of this town, on Tuesday, and the bequest, amounting to the large sum of £3,383, was handed over to Mr. Alderman Musgrave, J.P., who was accompanied by Mr. Alderman Nicholson, J.P. (one of the trustees of the infirmary), and Mr. Alderman Kevan (hon. secretary). According to the terms of the departed lady's will a further sum of £4,500 is bequeathed to the same institution, subject to the life interest of Miss Lowe's nephew, who is now 45 years of age. The following other gifts under Miss Lowe's will have been paid during the last few days : Blackpool Royal National Lifeboat Institution, £100 ; Lytham Lifeboat Institution, £100; St. Annes-on-the-Sea Lifeboat Institution, £100 ; Trustees Royal Albert Asylum, Lancaster, £500 ; Rivington Unitarian Chapel, £300. A sum of £1,000 is also bequeathed towards any scheme for an infirmary, dispensary, or cottage hospital for Blackpool."

SHERIFF DOVE-WILSON has felt compelled, owing to the increase in his judicial duties, to retire from the directorate of Aberdeen Infirmary, as he can no longer give the time necessary to the proper fulfilment of the duties connected with the position.

---

DURING the past year the patients at Richmond Hospital numbered—in-patients, 399 ; out-patients, 2,708—a considerable advance on the work done in 1868, when the in- atients numbered 115, and the out-patients 430. The committee are very glad to find that the working classes contribute so much by means of the collecting-boxes exhibited in various places. The income from this source alone amounted, during the year, to £313 11*s.* 7*d.*

---

MESSRS. LOFT AND BURTON, of Beverley, have decorated the board-room of the Beverley Cottage Hospital at their own cost, as a Jubilee offering. At the last annual meeting of the governors of this hospital two gentlemen offered themselves for the vacant post of auditor, and proposed to return the fees as a donation to the hospital. On these terms the directors appointed Mr. J. W. Arnott, steward of the East Riding Asylum, the other candidate being Mr. Pumfrett. The directors expressed their appreciation of the generosity shown by both gentlemen in making such an offer.

---

DR. JAMES RODGER, in his presidential address delivered to the Medico-Chirurgical Society of Aberdeen, gives an interesting account of the growth of the society. Like all institutions and individuals it had many trials in its infant days ; but since its commencement in 1789—the year of revolution, when several institutions were founded which have proved less permanent and valuable than the Aberdeen Society—it has been steadily growing in size and strength, and continues to provide a needed and valued means of professional intercourse for the medical men of "the granite city."

---

AT the annual meeting of the Wirral Children's Hospital, Birkenhead, special thanks were given to the lady collectors for the energy and success they had displayed in gaining subscriptions for the hospital. The ordinary income of the hospital was, however, insufficient to cover the expenditure ; but a special donation of £100 enabled the committee to show a balance of £1 7*s.* The governors expressed their regret that Mr. R. Marquis (the hon. treasurer) felt compelled to resign the office he had filled during the last four years, during which time he had done much for the hospital ; not merely in looking after the accounts, but in inducing people to subscribe. As a proof that Mr. Marquis despised no task that would contribute to the usefulness of the hospital, it may be mentioned that he took the part of Father Christmas at the recent entertainment to the patients, and sustained it to admiration.

---

THE latest addition to the number of Edinburgh charities bears the name of "Holiday House," and is intended to give a fortnight's residence in the country annually to as many children as its funds will permit. Those receive a preference who, though not ill, are yet not robust enough to stand the "roughing" that is almost inevitable when children are boarded-out in cottages, and which is not by any means bad for them when they are strong. "Holiday House" stands between these cottage homes and a convalescent institution, and the report of its one year of existence shows that it is doing work which is gratefully appreciated by those who benefit by it. It is very pleasant to hear how the children who have been in the house a week act as hosts to the new comers, and instruct them in all the rules of the place. "Holiday House" owes its existence mainly to Miss Chalmers, of Redhall, Slateford, a member of the family which has already given Edinburgh the "Chalmers Hospital."

